# Supplementary material for: Rutin-Loaded Poloxamer 407-Based Hydrogels for In Situ Administration: Stability Profiles and Rheological Properties
Source: Nanomaterials (Basel). 2020 May 31;10(6):1069. doi: 10.3390/nano10061069 (PMC7352531; doi:10.3390/nano10061069)
Supplement: Supplementary file 1 [file nanomaterials-10-01069-s001.pdf]

nanomaterials

Supplementary Materials

Rutin-loaded poloxamer 407-based hydrogels for *in situ* administration: stability profiles and rheological properties

Elena Giuliano [1], Donatella Paolino [2], Maria Chiara Cristiano [2], Massimo Fresta [1] and Donato Cosco [1,*]

[1] Department of Health Sciences, University "Magna Græcia" of Catanzaro, Campus Universitario "S. Venuta", Viale S. Venuta, I-88100 Catanzaro, Italy; giuliano.ele@gmail.com (E.G.); fresta@unicz.it (M.F.)
[2] Department of Experimental and Clinical Medicine, University "Magna Græcia" of Catanzaro, Campus Universitario "S. Venuta", Viale S. Venuta, I-88100 Catanzaro, Italy; paolino@unicz.it (D.P.); mchiara.cristiano@unicz.it (M.C.)
* Correspondence: donatocosco@unicz.it; Tel.: +39-0961-369-4119

Rutin	Poloxamer 407
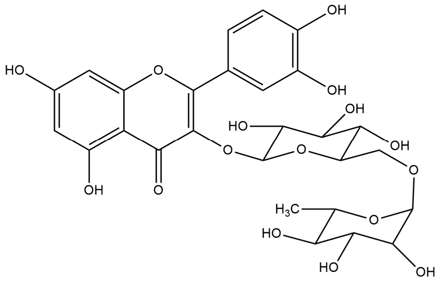	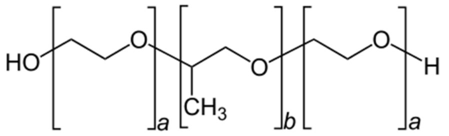 a = 101; b = 56

Figure S1. Chemical structure of rutin and poloxamer 407.

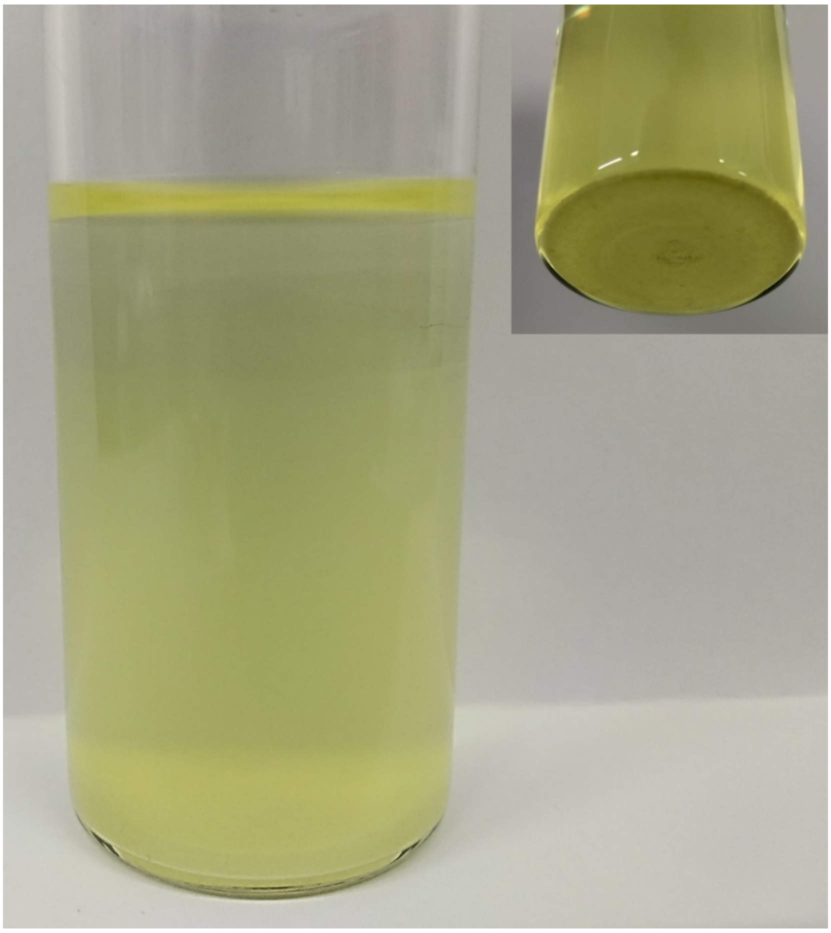

Figure S2. P407-based hydrogel prepared with 0.2% (w/w) of rutin

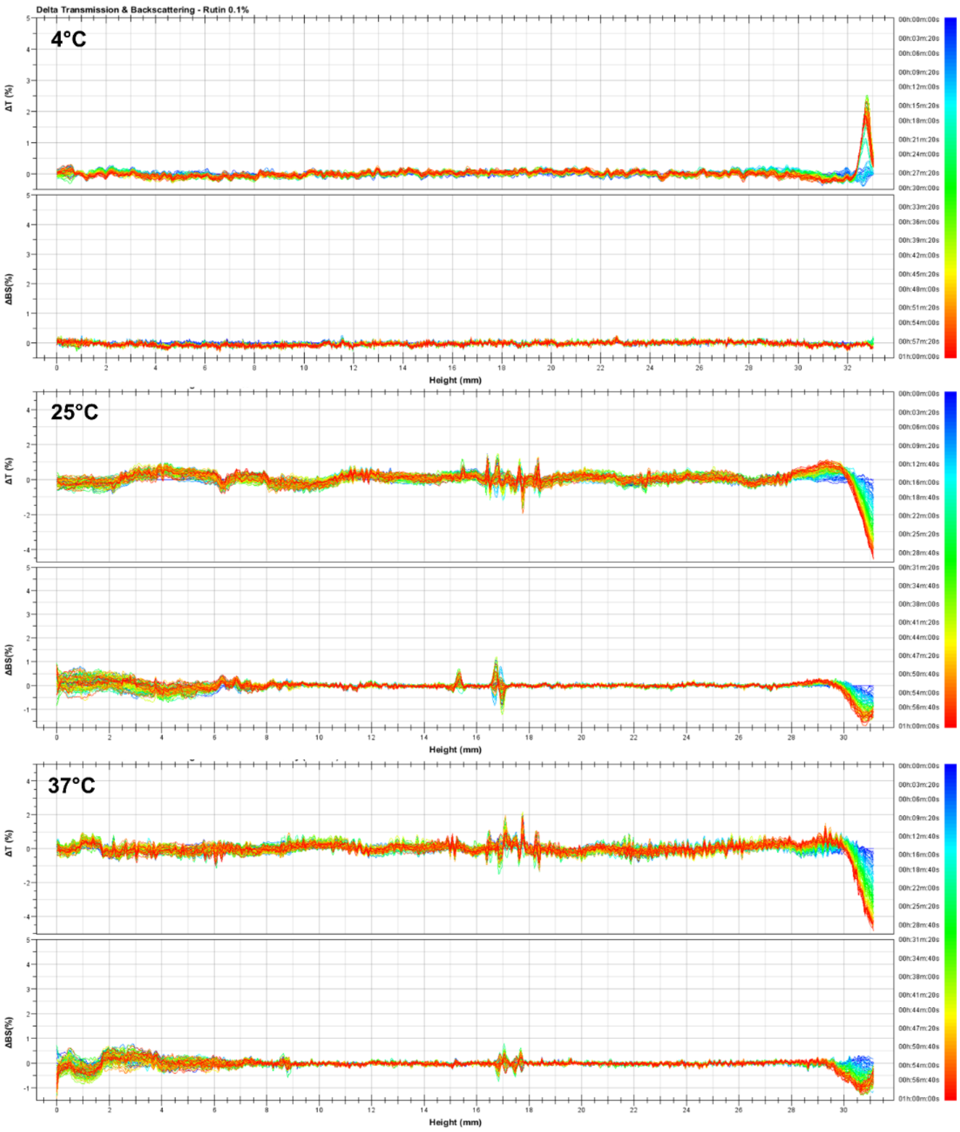

Figure S3. ΔBS and ΔT profiles of P407 hydrogels prepared with 0.1% (w/w) of rutin added in powder form as a function of the temperature and incubation time.

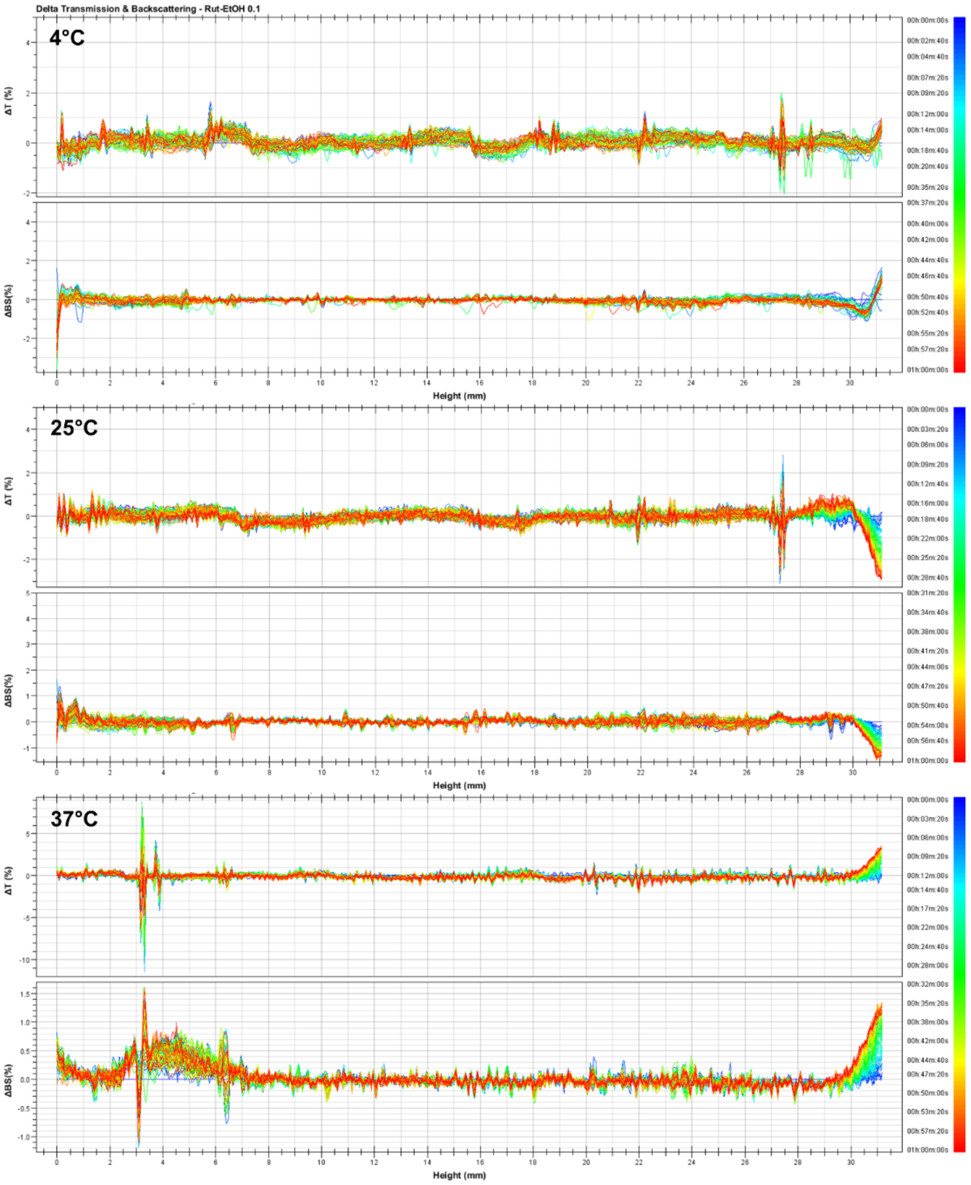

Figure S4. ΔBS and ΔT profiles of P407 hydrogels prepared with 0.1% (w/w) of rutin added as an ethanol solution as a function of the temperature and incubation time.

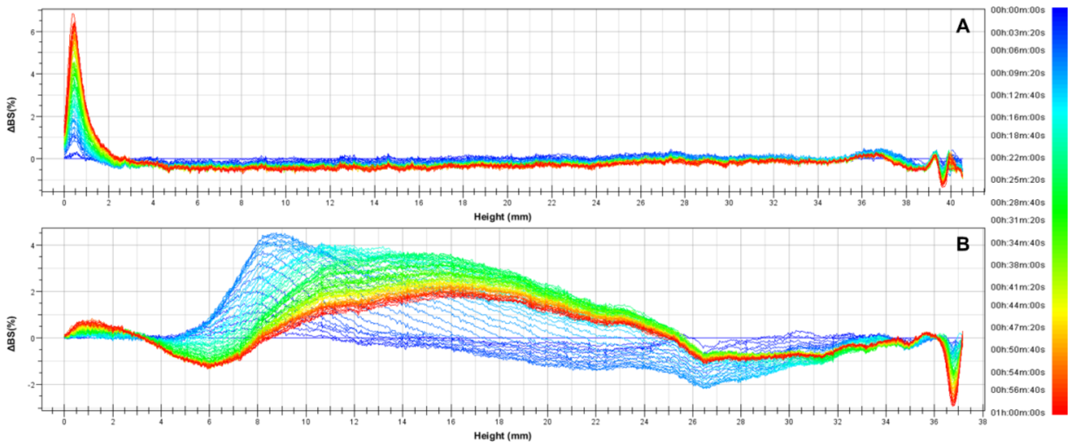

Figure S5. ΔBS profiles of rutin-loaded P407 hydrogels at 4 °C prepared using (A) 0.2% (w/w) and (B) 0.5% (w/w) of the drug. During the analysis a progressive increase of the ΔBS profiles occurred. This was due to the precipitation of the drug in the case of the formulation prepared with 0.2% of rutin and to the formation of macroaggregates in the system with 0.5% of the bioactive.

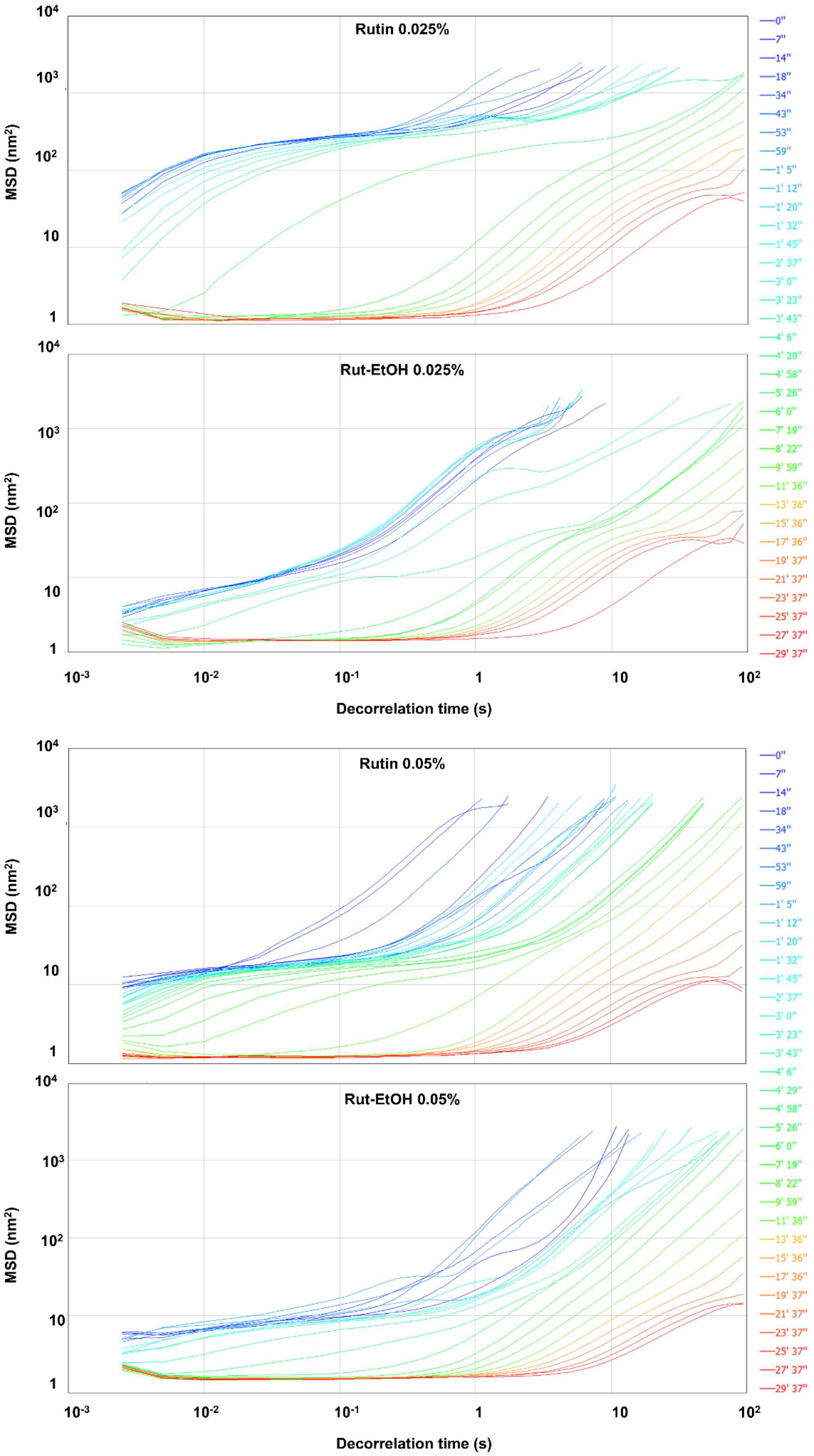
Rutin 0.025%
Rut-EtOH 0.025%
Rutin 0.05%
Rut-EtOH 0.05%
MSD (nm²)
Decorrelation time (s)
10⁻³
10⁻²
10⁻¹
1
10
10²
10³
10⁴
0"
7"
14"
18"
34"
43"
53"
59"
1' 5"
1' 12"
1' 20"
1' 32"
1' 45"
2' 37"
3' 0"
3' 23"
3' 43"
4' 6"
4' 29"
4' 58"
5' 26"
6' 0"
7' 19"
8' 22"
9' 59"
11' 36"
13' 36"
15' 36"
17' 36"
19' 37"
21' 37"
23' 37"
25' 37"
27' 37"
29' 37"

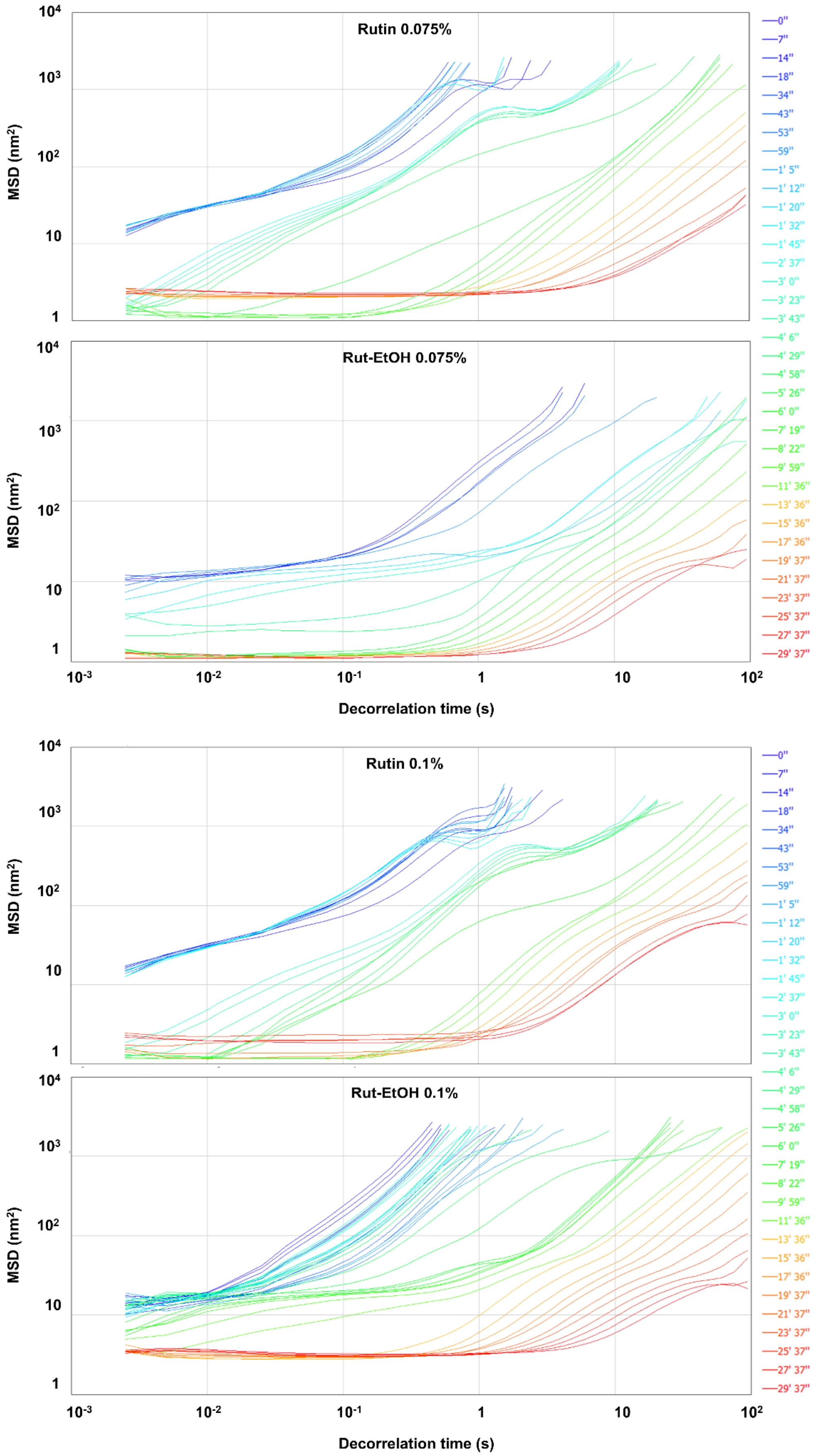

Figure S6. MSD curves of various P407 (20%, w/w)-based hydrogels containing different amounts of rutin as a powder or solubilized in ethanol as a function of the decorrelation time and temperature (from 4 °C to 37 °C).

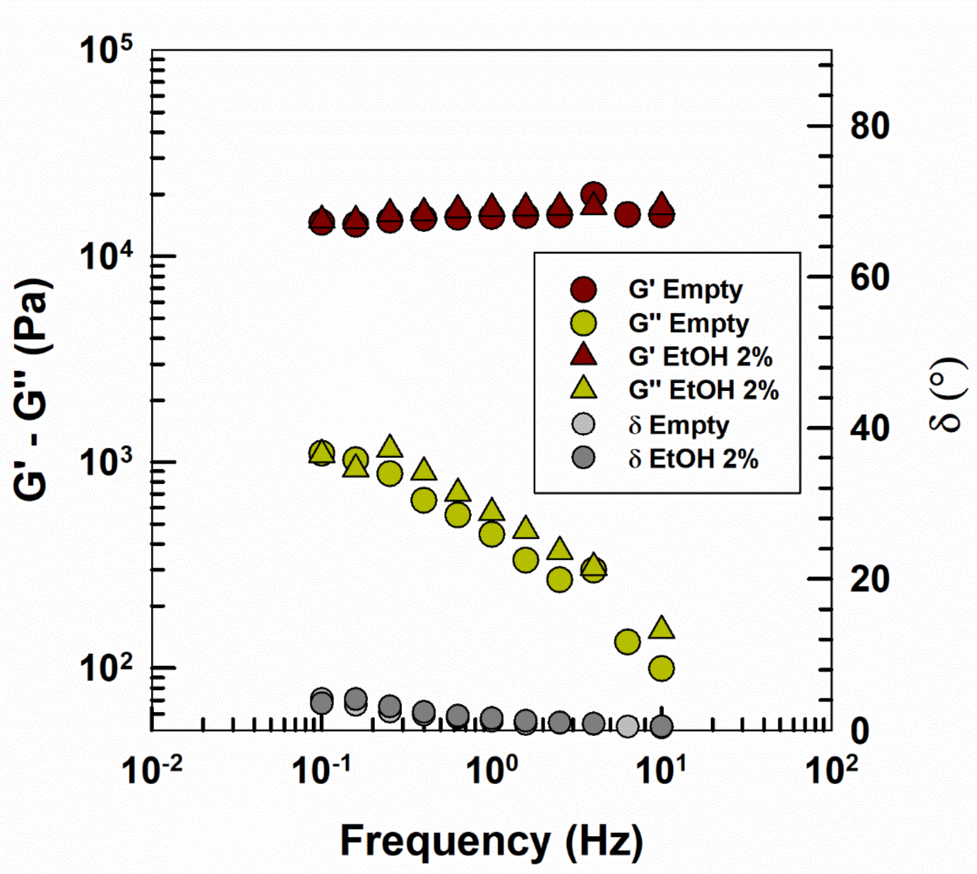

Figure S7. Elastic modulus (G'), viscous modulus (G'') and phase angle (δ) of P407 (20%, w/w) hydrogels as an ethanol-free system or containing the organic solvent (2%, w/w) as a function of frequency. This analysis was performed at 37 °C.

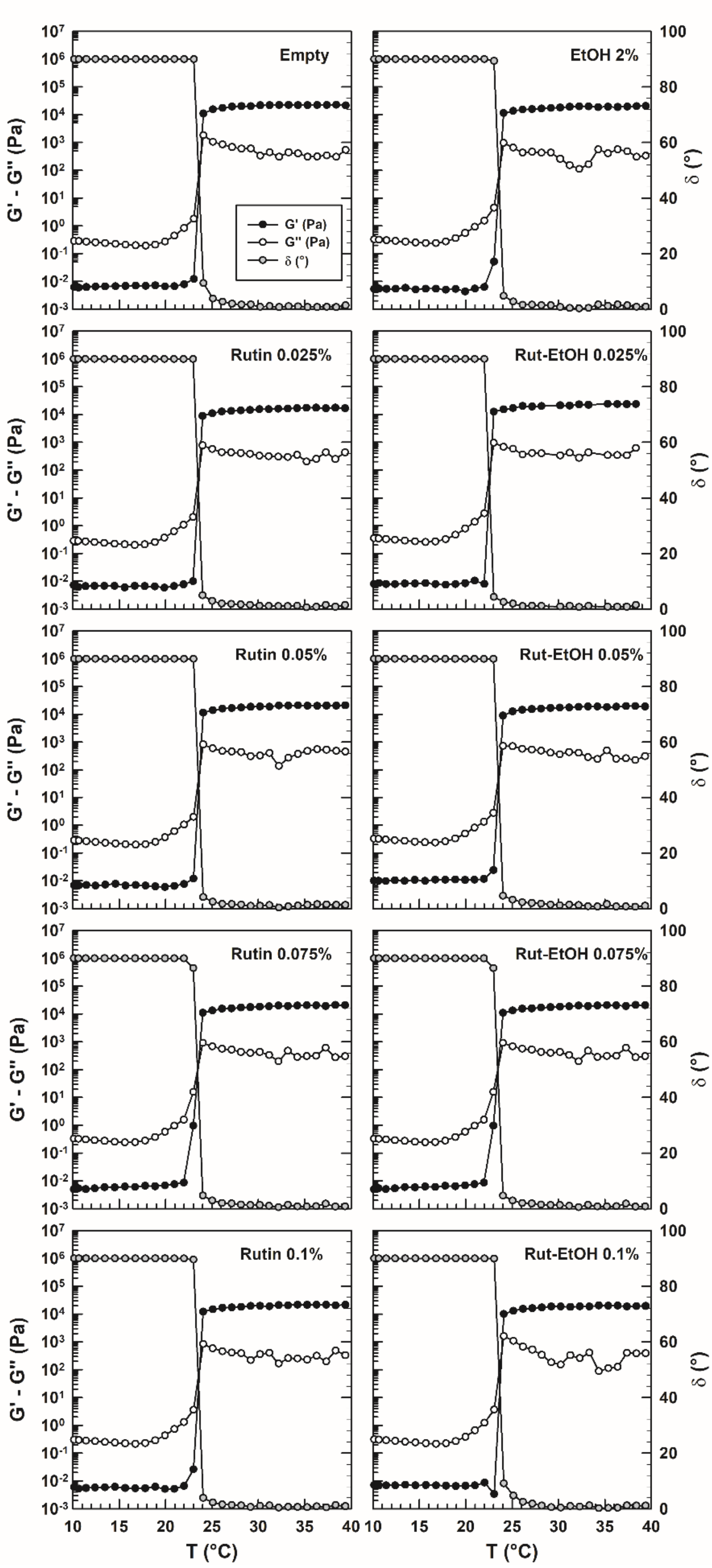

Figure S8. Elastic modulus (G′), viscous modulus (G′′) and phase angle (δ) of the various P407-hydrogels as a function of temperature.

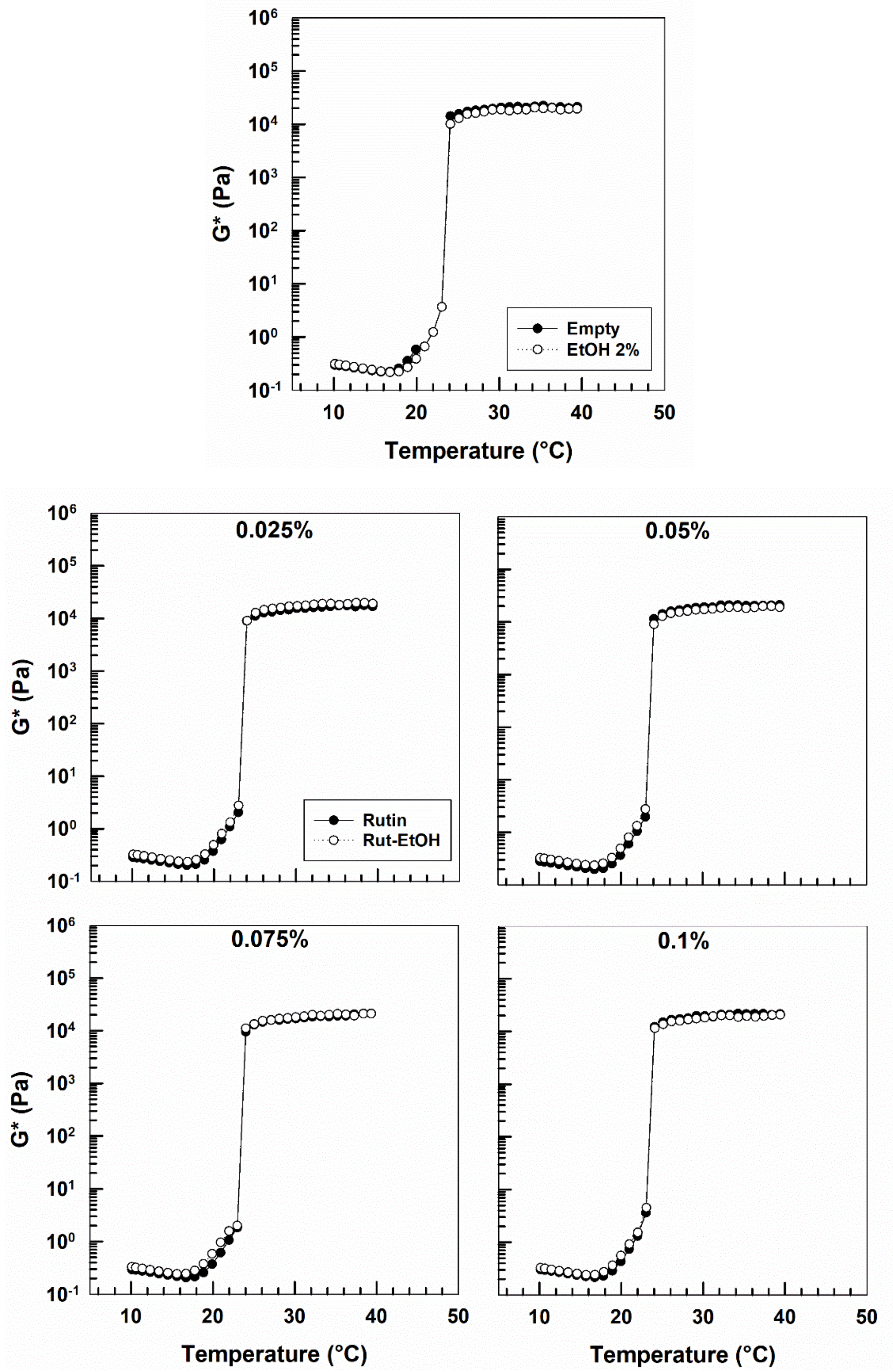

Figure S9. Complex modulus profiles of the various P407-hydrogels as a function of temperature.

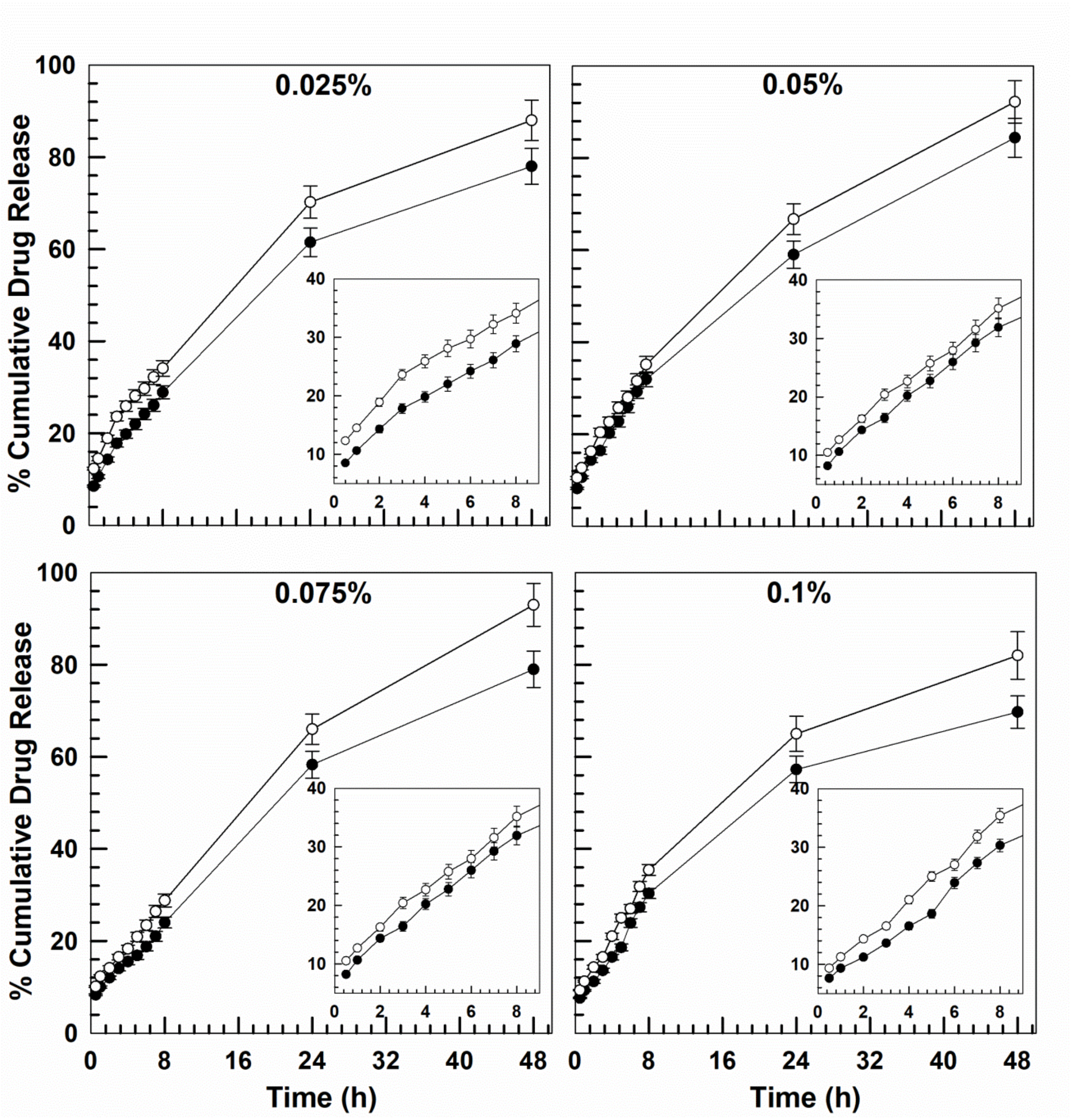

Figure S10. Release profiles of rutin in PBS from P407-based hydrogels. Experiments were carried out below the transition temperature. Values are the average of three different experiments ± standard deviation**.**

Table S1. Values of SLB (solid-liquid balance) of the of the various P407-based hydrogels.

Formulations	SLB t_i [1]	SLB t_f [1]
Empty	0.67	0.04
EtOH 2%	0.89	0.03
Rutin 0.025%	0.89	0.02
Rutin 0.05%	0.89	0.02
Rutin 0.075%	0.89	0.02
Rutin 0.1%	0.69	0.02
Rut-EtOH 0.025%	0.86	0.01
Rut-EtOH 0.05%	0.81	0.01
Rut-EtOH 0.075%	0.78	0.01
Rut-EtOH 0.1%	0.80	0.01

[1] t_i: initial time of the analysis; t_f: final time of the analysis

Table S2. Values of the phase angle (δ) of rutin-loaded P407 hydrogels as a function of frequency at 37 °C.

**Frequency (Hz)**	**Empty**	**EtOH 2%**	**Rutin 0.025%**	**Rutin 0.05%**	**Rutin 0.075%**	**Rut-EtOH 0.025%**	**Rut-EtOH 0.05%**	**Rut-EtOH 0.05%**	**Rut-EtOH 0.075%**	**Rut-EtOH 0.1%**
10.0	0.4	0.5	0.6	0.7	0.3	0.8	0.3	0.5	0.7	0.5
6.3	0.5	0.6	0.6	0.6	0.7	0.7	0.4	0.7	0.7	0.6
4.0	0.8	0.8	0.8	0.7	0.7	0.8	0.5	0.8	0.7	0.8
2.5	1.0	1.0	0.8	0.9	1.1	0.9	0.6	0.9	0.8	0.8
1.6	0.8	1.2	1.3	1.2	1.1	1.2	0.8	1.1	1.1	1.2
1.0	1.2	1.6	1.4	1.5	1.2	1.4	0.5	1.3	1.3	1.3
0.6	1.6	1.9	1.4	1.7	1.6	1.7	1.1	1.6	1.4	1.6
0.4	2.1	2.4	1.9	1.8	2.0	2.2	2.5	1.9	1.8	1.9
0.3	2.4	3.1	2.6	2.4	2.9	2.8	2.2	2.2	2.1	2.5
0.2	3.4	4.1	2.5	3.0	1.8	3.5	2.4	3.1	2.6	2.9
0.1	4.1	3.6	6.1	5.1	2	4.8	3.4	4.1	3.0	2.4

Abbreviations

P407	Poloxamer 407
EtOH	Ethanol
$T_{sol\text{-}gel}$	Solution-gel transition temperature
MLS	Multiple Light Scattering
ΔBS	Delta Back-scattering
ΔT	Delta Transmission
TSI	Turbiscan stability index
MS-DWS	Multi-speckle diffusing wave spectroscopy
MSD	Mean square displacement
SLB	Solid-liquid balance
EI	Elasticity index
LVR	Linear viscoelastic region
MW	Molecular weight
